# Association Between the Use of Angiotensin-Blocking Medications with Hip Fracture and Death in Older People

**DOI:** 10.14283/jfa.2019.38

**Published:** 2019-10-30

**Authors:** C. Shea, Miles D. Witham

**Affiliations:** 1 School of Medicine, University of Dundee, Dundee, UK; 2 AGE Research Group, NIHR Newcastle Biomedical Research Centre, Newcastle University and Newcastle upon Tyne Hospitals Trust, Newcastle, UK; 3 AGE Research Group, NIHR Newcastle Biomedical Research Centre, Newcastle University, NE4 5PL, Newcastle, UK

**Keywords:** Hip fracture, ACE inhibitor, angiotensin receptor blocker, mortality

## Abstract

It is unclear if angiotensin blocking drugs (angiotensin converting enzyme inhibitors and angiotensin receptor blockers) reduce or increase the risk of falls and fractures. We retrospectively analysed routinely-collected, linked health and social care data for patients aged 65 and over from Tayside, Scotland, including hospital discharge diagnoses, biochemistry, deaths, care package provision and community prescribing. We conducted unadjusted and adjusted Cox regression analyses for time to hip fracture and time to death, for any exposure to angiotensin blocking drugs and for time-dependent exposure to angiotensin blocking drugs. We analysed data on 16782 patients. Angiotensin blocking drug use was associated with an exposure-dependent lower risk of hip fracture (hazard ratio 0.988 [95%CI 0.982-0.994] per year of exposure; p<0.001) and death (hazard ratio 0.986 [95%CI 0.983–0.989] per year of exposure; p<0.001). These findings call into question the appropriateness of stopping angiotensin blocking drugs for older people at risk of falls.

## Introduction

Falls are a key consequence of the frailty syndrome, and hip fracture is a particularly feared consequence of falling. Controversy continues in the extent to which antihypertensive medication might contribute to falls risk; such medications are frequently stopped in older people who fall because of a perception that such medications increase falls risk by reducing blood pressure and hence cerebral perfusion. However some classes of medication, notably angiotensin-converting enzyme inhibitors (ACEi) and angiotensin receptor blockers (ARBs) have been shown to have beneficial effects on muscle function in preclinical and some clinical studies (1, 2, 3, 4). This raises the possibility that such medications could in fact be protective against falls, with recent systematic reviews supporting this possibility (5). There is currently uncertainty from observational studies as to whether ACEi and ARBs reduce or increase the risk of fractures, in part because analyses to date have not been able to adjust for functional status at baseline (6, 7, 8, 9, 10, 11, 12, 13). In addition, some studies have not been able to account for cumulative exposure to these medications, instead restricting analyses to baseline exposure with consequent indication bias. The aim of this analysis was therefore to test the association between ACEi / ARB use and hip fracture using a large, routinely collected, linked health and social care dataset.

## Methods

We performed a retrospective analysis of linked routinely-collected health and social care data. We studied patients aged 65 and over within the Tayside health board area, Scotland, UK (population 400,000), who had contact with Dundee city council social services between 2002 and 2012. The date of first social services assessment was taken as the inception date for each individual in the analysis cohort. We derived cumulative exposure to ACEi or ARB (in days) and any exposure including prior to the inception date from community prescribing records of prescriptions that were encashed with pharmacists. Deaths were obtained from Scottish General Register Office records. Hip fractures and comorbidity diagnoses were derived from hospital discharge records using ICD-10 codes. Hip fracture was diagnosed by the presence of ICD-10 codes S72.0, S72.1, S72.2, S72.8 or S72.9. Biochemical covariates were obtained from routinely collected biochemistry data analysed at Ninewells Hospital, Dundee. The need for a care package was used as a proxy for functional impairment; these data were obtained from Dundee City Council social work records. The methods used for sourcing and assembling these linked data have been described previously (14). All data were held with the Dundee Health Informatics Centre safe haven environment. Local Caldicott Guardian (data protection officer) approval was in place for all studies involving this linked dataset; the need for research ethics approval was waived as the analysis was performed using data collected as part of routine clinical care with no additional patient contact, which had then been anonymised prior to release to the research team.

We conducted unadjusted and adjusted Cox regression analyses for time to hip fracture and time to death, starting with any previous exposure to ACEi/ARB and then adding time-dependent exposure to ACEi/ARB. Such an approach allows the effect of drug exposure to be at least partly separated from the presence of the drug as a marker of underlying disease. Although hip fracture was our primary outcome of interest, we analysed all-cause mortality as well to ensure that any benefit on hip fracture was not counterbalanced by an adverse effect on mortality rates. We additionally conducted competing risks analysis using the method of Fine and Gray (15) to test whether accounting for all-cause mortality as a competing risk modified the association between ACEi/ARB use and hip fracture. Analyses were adjusted for baseline age, sex, deprivation, comorbidities, previous hip fracture, creatinine, bisphosphonate therapy, calcium and vitamin D therapy and receipt of a care package as a proxy for impaired physical function. These factors were chosen as they were available in linked, routinely collected clinical data and have all been shown to influence the risk of hip fracture or the risk of death. We also conducted exploratory subgroup analyses for age (above and below median for the study population), sex, and the presence of a package of social care as a surrogate for frailty. All analyses were conducted in SPSS v24 (IBM, New York, USA) with the exception of competing risks analyses, which were conducted using the stccreg function in STATA version 14 (STATAcorp, Texas, USA). A two-sided p value of <0.05 was taken as significant for all analyses.

## Results

A total of 16782 patients were included in the analysis. The mean age was 74.2 years and 9957 (59%) were female. 8354 (50%) had been exposed to ACEi/ARB therapy at any point prior to death, fracture or censoring. Table 1 shows the baseline details of the cohort. Users of ACEi/ARB were more likely to be male, had more comorbid disease, and were more likely to be in receipt of a care package than those who had never used ACEi or ARB. Table 2 shows the risk of hip fracture and of death in unadjusted and adjusted analyses. Analyses of any exposure to ACEi/ARB and time-dependent analyses accounting for cumulative exposure to ACEi/ARB are presented, showing that in adjusted analyses, cumulative exposure to ACEi or ARB was associated with an exposure-dependent reduction in the risk of hip fracture, and that this effect was in addition to the lower risk of hip fracture seen in those that had previously used ACEi or ARB. Similar, but more marked effects were seen for all-cause mortality. Unadjusted competing-risks models showed a hazard ratio for hip fracture of 0.992 (95%CI 0.986 to 0.998; p=0.006) per year of exposure to ACEi/ARB; in adjusted competing-risks analyses, the effect did not reach significance (HR 0.996; 95%CI 0.991 to 1.002; p=0.21).

**Table 1:** Baseline details of analysis cohort

	Never on ACEi or ARB n=8428	ACEi or ARB use n=8354	P
Mean age (years) (SD)	73.5 (9.4)	73.8 (8.4)	0.05
Female (%)	5146 (61.1)	4811 (57.6)	<0.001
Decile of deprivation (SD)	5.4 (3.0)	5.0 (3.0)	<0.001
Previous myocardial infarction (%)	257 (3.0)	1189 (14.2)	<0.001
Previous stroke (%)	307 (3.6)	733 (8.8)	<0.001
Congestive heart failure (%)	219 (2.6)	761 (9.1)	<0.001
Chronic obstructive pulmonary disease (%)	546 (6.5)	609 (7.3)	0.038
Diabetes mellitus (%)	506 (6.0)	1730 (20.7)	<0.001
Previous hip fracture (%)	317 (3.8)	225 (2.7)	<0.001
Mean creatinine (µmol/l) (SD)	91 (55)	101 (61)	<0.001
Bisphosphonate therapy (%)	379 (4.5)	397 (4.8)	0.43
Calcium / Vitamin D therapy (%)	505 (6.0)	566 (6.8)	0.038
Current care package (%)	543 (6.4)	676 (8.1)	<0.001
Died during follow up (%)	2534 (30.1)	1699 (20.3)	<0.001
New hip fracture (%)	390 (4.6)	255 (3.1)	<0.001


ACEi: Angiotensin converting enzyme inhibitor. ARB: Angiotensin receptor blocker

**Table 2:** Cox proportional hazards models showing effect of ACEi / ARB exposure on risk of hip fracture or death

		Hip fracture Hazard ratio (95% CI)	P	All-cause death Hazard ratio (95% CI)	P
Previously exposed					
ACEi or ARB exposure — unadjusted analysis		0.760 (0.635–0.910)	0.003	0.968 (0.906–1.033)	0.32
ACEi or ARB exposure — adjusted analysis*		0.707 (0.585–0.853)	<0.001	0.797 (0.743–0.855)	<0.001
Cumulative exposure (time-dependent analysis)					
Unadjusted analysis	ACEi or ARB exposure (per year of exposure)	0.983 (0.977–0.990)	<0.001	0.982 (0.979–0.985)	<0.001
	Ever exposed to ACEi or ARB	0.903 (0.747–1.091)	0.29	1.081 (1.010–1.157)	0.024
Adjusted analysis	ACEi or ARB exposure (per year of exposure)	0.988 (0.982–0.994)	<0.001	0.986 (0.983–0.989)	<0.001
	Ever exposed to ACEi or ARB	0.788 (0.647–0.959)	0.017	0.862 (0.802–0.926)	<0.001


*adjusted for age, sex, deprivation, comorbidities, previous hip fracture, creatinine, bisphosphonate therapy, calcium and vitamin D therapy, receipt of care package; ACEi: Angiotensin converting enzyme inhibitor. ARB: Angiotensin receptor blocker

Exploratory subgroup analyses for age above or below the median (74 years), sex, or the presence or absence of a package of care are shown in Table 3. The hazard ratio for death per year of exposure to ACEi/ARB was lower for those receiving a package of care, and this difference was significant (p=0.03) on formal interaction testing; no other significant differences were seen between subgroups.

**Table 3:** Exploratory subgroup analyses — effect of cumulative exposure in adjusted time-dependent analyses

		Hip fracture Hazard ratio (95% CI)*	P	All-cause death	
Package of care	Yes	0.988 (0.973–1.004	0.13	0.979 (0.960–0.998)	0.032
	No	0.987 (0.981–0.994)	<0.001	0.986 (0.983–0.989)	<0.001
Age	>=74 years	0.990 (0.981–0.998)	0.014	0.985 (0.981–0.990)	<0.001
	<74 years	0.983 (0.974–0.992)	<0.001	0.986 (0.982–0.990)	<0.001
Sex	Male	0.978 (0.962–0.994)	0.008	0.986 (0.981–0.991)	<0.001
	Female	0.990 (0.984–0.997)	0.003	0.986 (0.982–0.990)	<0.001


Adjusted for age, sex, deprivation, comorbidities, previous hip fracture, creatinine, bisphosphonate therapy, calcium and vitamin D therapy, receipt of care package with the exception of the subgroup variable under test; *Hazard ratio per year of ACEi/ARB exposure

## Discussion

In this analysis, exposure to ACEIs/ARBs was associated with an exposure-dependent reduction in the risk of hip fractures and death. The analysis sample reflects a real-world sample due to the use of routinely collected data, was able to use time-dependent exposure to medication, and was also able to adjust for a range of comorbidities, biomarkers, and importantly, was able to adjust for daily function using a proxy measure (the need for social care). The results are in accord with the majority of previous observational studies (6, 7, 8, 9, 10, 11, 12, 13), and are also supported by findings from the Hypertension in the Very Elderly (HYVET) trial, which found a lower rate of fractures in older people with hypertension treated with thiazides and ACE inhibitors (16), but differ from a post-hoc analysis of the Antihypertensive and Lipid Lowering to prevent Heart Attack (ALLHAT) trial, where ACEi did not confer a lower risk of fracture compared to calcium channel blockers, and were inferior to thiazides (17). Given the known beneficial effects of thiazides on bone mineral density, these results are not incompatible however. Recent observational data from the Womens Health Initiative studies suggests variable risk with time; short-term increased risks may be balanced by longer-term benefits to ACEi use (18).

ACE inhibitors may reduce fracture rates via a range of different biological mechanisms. Although such medications are often stopped in patients with orthostatic hypotension, the evidence that modern, long-acting ACEi and ARBs cause orthostatic hypotension is very limited (19); the underlying vascular disease that such agents are used to treat is as likely to explain any such association in observational studies. On the contrary, ACEi and ARB are known to improve endothelial function and vascular stiffness (20, 21), and thus could in fact mediate improvements in vascular tone and control that would mitigate orthostatic hypotension. ACEi have also been postulated to directly improve skeletal muscle function; observational data suggests that those taking ACEi exhibit slower declines in walk speed, and 20 weeks of ACEi therapy improved six-minute walk distance in older people with functional impairment (2). ACEi do not appear to improve postural sway in older people at risk of falls however (4). Finally, it is possible that ACEi and ARB may have beneficial effects on bone mineral density, although observational studies to date suggest that this benefit may be confined to African-American men (22, 23).

Observational studies cannot ascertain causality, even with adjustment for multiple confounders, and our findings may still be due to residual confounding. We could not adjust directly for frailty, but relied on a surrogate measure of functional impairment. We did not compare the effect of ACEi/ARB use with use of other classes of antihypertensive medication, although dissecting out effects for different classes in patients on multiple medications would be challenging. We were also unable to adjust for some important covariates, such as blood pressure, as these data were not available in electronic form in the routinely collected datasets available for this analysis. Similarly, data on cognition were not available in electronic form, and would not have been recorded as part of routine care for many of those studied in this analysis. Some diagnoses, such as a diagnosis of hypertension, are not well recorded on hospital discharge codes and we could not therefore adjust for such covariates. Despite these limitations, our findings, in conjunction with previous observational and trial evidence, at least call into question the appropriateness of avoiding or discontinuing ACEi/ARBs in older people at risk of falls and fracture.

*Funding:* Chief Scientist Office, Scottish Government, grant number SCPH/10

*Ethical standards:* This analysis used anonymised, routinely collected healthcare data and thus did not require separate ethics approval. Please see text for details.

*Conflicts of interest:* The authors declare that they have no conflicts of interest

## References

[1] Onder G, Penninx BW, Balkrishnan R (2002). Relation between use of angiotensin-converting enzyme inhibitors and muscle strength and physical function in older women: an observational study. Lance.

[2] Sumukadas D, Witham M, Struthers AD, McMurdo MET (2007). Effect of perindopril on physical function in elderly people with functional impairment: a randomized control trial. CMAJ.

[3] Cesari M, Pedone C, Incalzi RA, Pahor M (2010). ACE-inhibition and physical function: results from the Trial of Angiotensin-Converting Enzyme Inhibition and Novel Cardiovascular Risk Factors (TRAIN) study. J Am Med Dir Assoc.

[4] Sumukadas D, Price R, McMurdo MET (2018). The effect of perindopril on postural instability in older people with a history of falls—a randomised controlled trial. Age Ageing.

[5] Ang HT, Lim KK, Kwan YH (2018). A systematic Review and meta-analyses of the association between anti-hypertensive classes and the risk of falls among older adults. Drugs Aging.

[6] Torstensson M, Hansen AH, Leth-Møller K (2015). Danish register-based study on the association between specific cardiovascular drugs and fragility fractures. BMJ Open.

[7] Rejnmark L, Vestergaard P, Mosekilde L (2006). Treatment with beta-blockers, ACE inhibitors, and calcium-channel blockers is associated with a reduced fracture risk: a nationwide case-control study. J Hypertens.

[8] Butt DA, Mamdani M, Gomes T (2014). Risk of osteoporotic fractures with angiotensin II receptor blockers versus angiotensin-converting enzyme inhibitors in hypertensive community-dwelling elderly. J Bone Mineral Res.

[9] Kwok T, Leung J, Barrett-Connor E, Osteoporotic Fractures in Men MrOS Research Group (2017). ARB users exhibit a lower fracture incidence than ACE inhibitor users among older hypertensive men. Age Ageing.

[10] Ruths S, Bakken MS, Ranhoff AH, Hunskaar S, Engesæter LB, Engelan A (2015). Risk of hip fracture among older people using antihypertensive drugs: a nationwide cohort study. BMC Geriatr.

[11] Chen HY, Ma KY, Hsieh PL, Liou YS, Jong GP (2016). Long-term effects of antihypertensive drug use and new-onset osteoporotic fracture in elderly patients: a population-based longitudinal cohort study. Chin Med J.

[12] Solomon DH, Mogun H, Garneau K, Fischer MA (2011). Risk of fractures in older adults using antihypertensive medication. J Bone Mineral Res.

[13] Cheng YZ, Huang ZZ, Shen ZF (2017). ACE inhibitors and the risk of fractures: a meta-analysis of observational studies. Endocrine.

[14] Basu U, Goodbrand J, McMurdo MET (2016). Association between allopurinol use and hip fracture in older patients. Bone.

[15] Fine JP, Gray R (1999). A proportional hazards model for the subdistribution of a competing risk. J Am Statistical Assoc.

[16] Peters R, Beckett N, Burch L (2010). The effect of treatment based on a diuretic (indapamide) ± ACE inhibitor (perindopril) on fractures in the Hypertension in the Very Elderly Trial (HYVET). Age Ageing.

[17] Barzilay JI, Davis BR, Pressel SL (2017). The impact of antihypertensive medications on bone mineral density and fracture risk. Curr Cardiol Rep.

[18] Carbone L.D., Vasan S., Prentice R.L., Harshfield G., Haring B., Cauley J.A., Johnson K.C. (2019). The renin-angiotensin aldosterone system and osteoporosis: findings from the Women's Health Initiative. Osteoporosis International.

[19] Frith J, Parry SW (2017). New Horizons in orthostatic hypotension. Age Ageing.

[20] Virdis A, Ghiadoni L, Taddei S (2011). Effects of antihypertensive treatment on endothelial function. Curr Hypertens Rep.

[21] Koumaras C, Tziomalos K, Stavrinou E (2014). Effects of renin-angiotensin-aldosterone system inhibitors and beta-blockers on markers of arterial stiffness. J Am Soc Hypertens.

[22] Solomon DH, Ruppert K, Zhao Z (2016). Bone mineral density changes among women initiating blood pressure lowering drugs: a SWAN cohort study. Osteoporos Int.

[23] Rianon N, Ambrose CG, Pervin H (2017). Long-term use of angiotensin-converting enzyme inhibitors protects against bone loss in African-American elderly men. Arch Osteoporos.

